# Supplementary material for: Taurine Administration Counteracts Aging-Associated Impingement of Skeletal Muscle Regeneration by Reducing Inflammation and Oxidative Stress
Source: Antioxidants (Basel). 2022 May 21;11(5):1016. doi: 10.3390/antiox11051016 (PMC9137670; doi:10.3390/antiox11051016)
Supplement: Supplementary file 1 [file antioxidants-11-01016-s001.zip › antioxidants-1668028-supplementary.pdf]

Supplemental Materials

Materials and Methods

ROS determination

For determination of ROS production in skeletal muscle sections, frozen TA cross-sections were washed with ice-cold PBS for 5 min, incubated with 300 nM CM-H2DCFDA (C6827; Invitrogen) in PBS at 37° C for 30 min, and washed again in ice-cold PBS to stop the reaction. The sections were mounted with ProLong™ Gold Antifade Mountant with DAPI (Thermo Fisher Scientific, Waltham, MA, USA, #P36935), examined with an Olympus BX53 fluorescent microscope (Olympus, Tokyo, Japan), and captured with ISCapture software (Tucsen Photonics, Fujian, China). Quantification of the changes in the CM-H2DCFDA signal in the experimental groups was performed by densitometric analyses. After background subtraction, CM-H2DCFDA fiber-associated signals were quantified by manually outlining individual fibers and measuring fiber-associated fluorescence intensity with the ImageJ software. The F/A ratio defines the mean fluorescence of individual fibers (F) normalized to total fiber cross-sectional area (A). Quantification was done on 50 fibers per group (n=3 mice per group).

Results

Taurine attenuates ROS accumulation in TA muscles of aged mice

To confirm this effect of taurine we next examined in the TA of young, old, and old + taurine mice, ROS accumulation by using CM-H2DCFDA fluorescent probe as a useful indicator that may react with several ROS including singlet oxygen, superoxide, hydroxyl radical, and various peroxide [67]. Quantitative analysis performed by fluorescence microscopy demonstrated that the TA muscle of old mice displayed higher ROS levels compared to young and the presence of taurine downregulated the ROS accumulation in TA of old mice (Figures 1A and B).

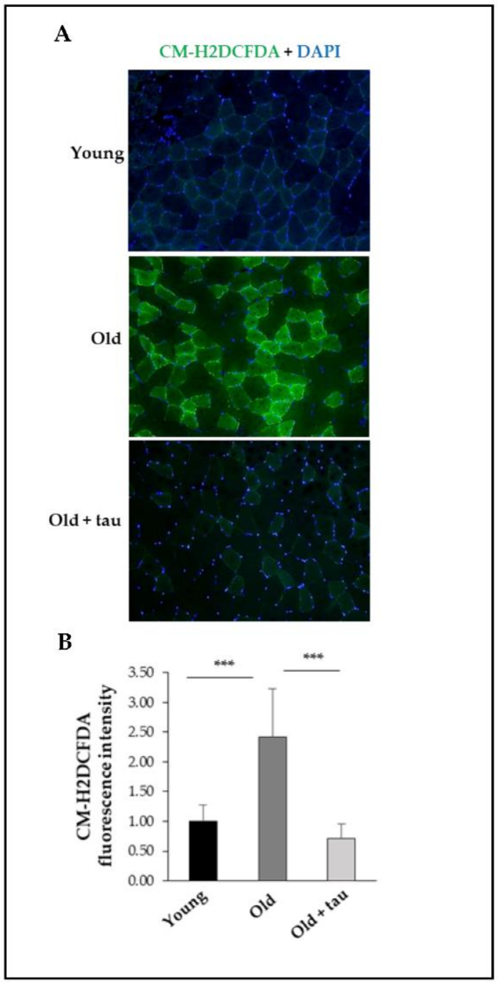

Figure S1: (A) Representative micrographs of TA cross-sections showing ROS levels assessed using CM-H2DCFDA and (B) quantification of fluorescence intensity. Statistical analysis was performed using one way ANOVA multiple comparison, *** $p < 0.001$, $n = 3$ mice per group.
